# Fried Soybean Oil Causes Systemic Low-Grade Inflammation by Disrupting the Balance of Gut Microbiota in Mice

**DOI:** 10.3390/microorganisms12061210

**Published:** 2024-06-16

**Authors:** Lianhua Hu, Ling Huang, Zhijia Fang, Chen Wang, Jinjin Luo, Qi Deng, Defeng Xu, Lijun Sun, Ravi Gooneratne

**Affiliations:** 1 Guangdong Provincial Key Laboratory of Aquatic Product Processing and Safety, College of Food Science and Technology, Guangdong Ocean University, Zhanjiang 524088, China; hu-lian-hua@163.com (L.H.); 2112203048@stu.gdou.edu.cn (L.H.); 18642326533@163.com (C.W.); 2112203018@stu.gdou.edu.cn (J.L.); gdoudengqi@163.com (Q.D.); 13827198525@163.com (D.X.); suncamt@126.com (L.S.); 2 Department of Wine, Food and Molecular Biosciences, Faculty of Agriculture and Life Sciences, Lincoln University, P.O. Box 85084, Lincoln 7647, New Zealand; ravi.gooneratne2@lincolnuni.ac.nz

**Keywords:** fried oils, short-term intake, systemic low-grade inflammation, gut microbiota, short-chain fatty acids

## Abstract

Previous reports have mainly investigated the long-term effects (>30 d), such as gut microbiota dysbiosis and systemic low-grade inflammation, in mice fed fried oil. However, short-term intake of deep-fried oil is more likely to occur in daily life, and such studies are lacking. This study aimed to investigate the short-term effects of fried oil intake on systemic low-grade inflammation. Male Kunming mice were fed non-fried soybean oil or low (25%), medium (50%), or high (100%)—fried oil at 4.4 g/kg for 6 d. Serum and fecal samples were collected on day 7. In all groups fed fried oil, the serum levels of tumor necrosis factor (TNF-α) were significantly elevated 2-4-fold. Among the gut microbiota, the abundance of *Alloprevotella* significantly decreased by up to 76%, while *Lactobacilli* significantly increased by up to 385%. The fecal valeric acid content was significantly increased and positively correlated with TNF-α levels. Both valeric acid and TNF-α levels were positively correlated with the abundance of *Lactobacilli* and negatively correlated with that of *Alloprevotella*. In summary, a short-term ingestion of even low doses of fried oil alters the gut microbiota *Alloprevotella* and *Lactobacilli* and increases fecal valeric acid content, which correlates with increased serum TNF-α levels.

## 1. Introduction

Many consumers favor fried food because of its crisp taste, quick preparation, and convenience. To reduce costs, reusing fried oil has become a common practice in many fast-food restaurants. Cooking oil is easily oxidized by frequent heating to high temperatures, which not only reduces its nutritional value but also can form toxic chemicals, including benzopyrene and heterocyclic amines, that can leach into food. Eating oil-fried food can lead to obesity, metabolic disorders, systemic low-grade inflammation, and other diseases [1,2].

The consumption of fried food commonly induces systemic low-grade inflammation. This state is non-specific, persistent, and mild, as evidenced by a 2-4-fold increase in plasma inflammatory factors such as TNF-α [3], a cytokine considered to be a key factor in age-related diseases, autoimmune disorders, cancer, cardiovascular diseases, and diabetes [4]. Therefore, it is important to investigate the low-grade inflammation mechanism caused by the repeated use of oil for frying to understand the mechanistic targets and their regulation.

Some studies suggest that the damage caused by fried oil to the body systems is a result of oxidative stress [5]. However, there is now strong evidence that the gut microbiota and its metabolic disturbances are also responsible for the induction of systemic low-grade inflammation, especially by changes in the composition and levels of short-chain fatty acids (SCFAs), which are mostly the metabolic end products of the gut microbiota [6]. They regulate body inflammatory responses by inducing or inhibiting cytokine production [7]. However, the changes in the microbiota and associated metabolite composition caused by different inducers and biomarkers of systemic low-grade inflammation are different. Therefore, clarifying the effects of fried oil on gut microbiota structure and SCFA levels is important for identifying the mechanisms by which fried oil induces systemic low-grade inflammation. Based on this, we hypothesized that a short-term consumption of repeatedly fried soybean oil would induce systemic low-grade inflammation by altering the structure of the gut microbiota community and the production of SCFAs, thus providing a basis for exploring targeted interventions to mitigate the health risks associated with a frequent consumption of fried foods.

The current literature on the harm caused by fried oil is mostly based on a long-term (≥30 days) and excessive consumption of fried oil [8,9], and there is a lack of research on the effects of short-term (one week) intake of fried oil. The short-term consumption of fried oil aligns with the frequency of the individual consumption of fried foods in daily life. Investigating the mechanism of induction of systemic low-grade inflammation following short-term exposure may offer more precise health recommendations.

This study explored the mechanism of low-grade inflammation induced by the short-term dietary intake of fried oil to provide theoretical support for exploring specific control targets.

## 2. Materials and Methods

### 2.1. Preparation of Fried Oil

Chicken fillets, French fries, and dough were fried in fresh soybean oil for 10 min each hour for 8 h (from 9 a.m. to 5 p.m.) for 4 consecutive days, for a total of 32 times. Four sets of soybean-fried oils were prepared based on the percentage of heated soybean oil in the mix. We used unheated soybean oil (C), 25% fried soybean oil +75% unheated soybean oil (low dose: LD), 50% fried soybean oil +50% unheated soybean oil (medium dose: MD), and 100% fried soybean oil (high dose: HD).

### 2.2. Animal Study

Six-week-old male Kunming mice (30 ± 2 g) were purchased from Changsha Tianqin Biotechnology Co., Ltd., Changsha, China (SPF, SCXK [Xiang] 2019-0014). The experimental animals were maintained at room temperature (RT: 25 ± 2 °C), a humidity of 40–70%, and a 12 h light/dark cycle. The experimental animals were approved by the Animal Ethics Committee of Guangdong Ocean University (GDOU-LAE-2020-018) and raised in strict accordance with the operating standards of the Experimental Animal Center of Guangdong Ocean University (SYXK2014-0053). The standard polypropylene cages used in the experiments were 53 cm in length, 39 cm in width, and 20 cm in height, which provided ample living space for the mice and complied with animal welfare and ethical standards. Water bottles, cages, and bedding materials were sterilized using high-pressure steam, and the water bottles and bedding materials were changed three times a week. After one week of adaptive feeding, 20 mice were randomly divided into 4 groups with 5 animals each: C, LD, MD, and HD. All mice received an intragastric administration of either unheated soybean oil (C) or fried oil (LD, MD, HD) at 4.4 g/kg, respectively, once daily for 6 d. The body weights of the mice were measured daily during the experimental period.

### 2.3. Serum TNF-α

After 6 days of gavage, blood was collected from the eyeballs of mice on day 7, and centrifuged at 4 °C, 8300 r/min, for 5min in a cryo-centrifuge (ST16R, Thermo Fisher Technology [China] Co., Ltd., Shanghai, China). The supernatant was aspirated and placed in a −80 °C ultra-low-temperature refrigerator (JW-86-158-LA). Serum TNF-α concentration was measured using a TNF-α ELISA kit (Shenzhen Xinbosheng Biotechnology Co., Ltd., Shenzhen, China) procedure.

### 2.4. Gut Microbiota

On day 7, the fecal samples of mice were collected into 1.5 mL sterile EP tubes and stored in an ultra-low-temperature refrigerator at −80 °C. Fecal samples were sent to Hangzhou GH Information Technology Co., Ltd., Hangzhou, China. for 16S rRNA gene sequencing and detection using an Illumina HiSeq4000 pair-end 2 × 150 bp sequencing platform. Gut microbiota strain categories were identified by database comparisons.

### 2.5. Fecal SCFAs

Fecal SCFA levels were detected using GC-MS (GCMS-TQ8050 NX, Shimadzu Co., Ltd., Kyoto City, Japan). Fecal samples (50 mg) were added to 100 μL of 15% phosphoric acid, followed by 100 μL of a 125 μg/mL isocaproic acid solution and 900 μL diethyl ether, and then homogenized and centrifuged for 10 min. The mixed solution was filtered using a 0.22 μm organic microporous membrane. The GC conditions were as follows: a VF-17MS capillary column; inlet temperature of 250 °C; ion source temperature of 230 °C; transmission line temperature of 250 °C; and four-bar temperature of 150 °C. MS conditions were the following: an electron bombardment ionization source, single-ion scanning mode, and electron energy of 70 eV.

### 2.6. Statistical Analysis

The experimental data were statistically analyzed using SPSS27.0 software. Pearson’s method was used to analyze data correlation. Data are expressed as the “mean ± SD”, and a one-way analysis of variance was used to compare the means of each group, with *p* < 0.05 indicating a statistically significant difference.

## 3. Results

### 3.1. Effects of Fried Oil on Body Weights

**Figure 1:**
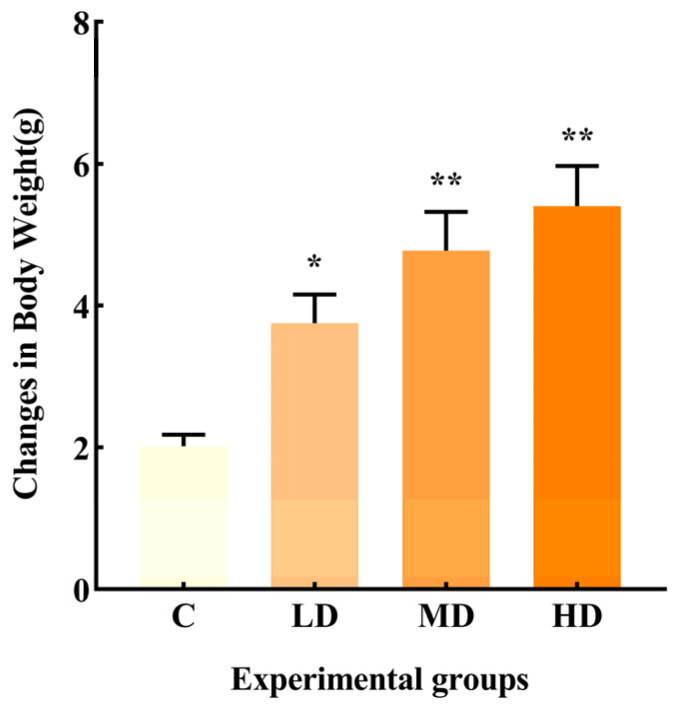
Effects of different doses of fried oil on body weight of mice after six days of intragastric administration. Data are expressed as mean ± SD. * *p* < 0.05, ** *p* < 0.01 vs. C.

As shown in Figure 1, the change in body weight in the LD, MD, and HD groups was significantly higher than that in group C, with increases of 86.3% (*p* < 0.05), 137%, and 168% (*p* < 0.01), respectively.

### 3.2. Effects of Fried Oil on Serum TNF-α

**Figure 2:**
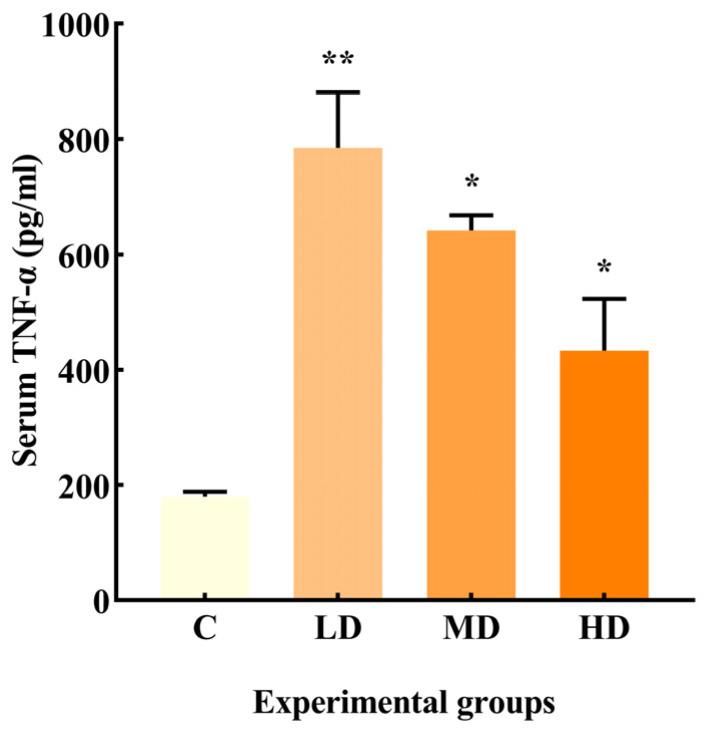
Effects of different doses of fried oil on serum TNF-α in mice. Data are expressed as mean ± SD. * *p* < 0.05, ** *p* < 0.01 vs. C.

As illustrated in Figure 2, the LD, MD, and HD groups exhibited a significant increase in TNF-α production by 335% (*p* < 0.01) and 256% and 140% (*p* < 0.05), respectively, compared to the C group.

### 3.3. Effects of Fried Oil on the Gut Microbiota Diversity

**Figure 3:**
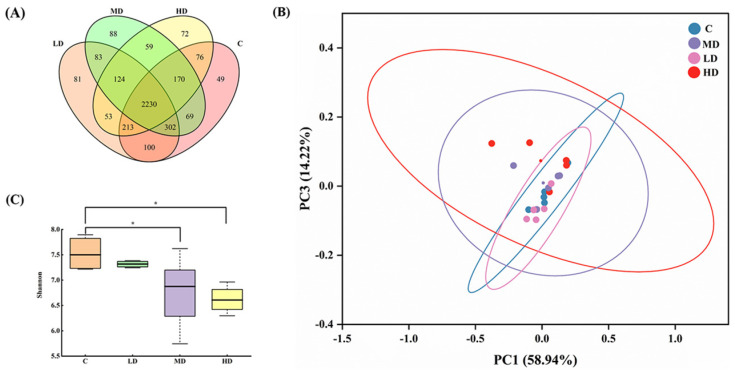
Effects of fried oil on gut microbiota diversity in mice. (**A**) Venn diagram display of mouse gut microbiota in each group. (**B**) Principal coordinate analysis showing mouse gut microbiota structure. (**C**) Shannon index of mouse gut microbiota. Data are expressed as mean ± SD. * *p* < 0.05 vs. C.

As shown in Figure 3A, 3209, 3186, 3125, and 2997 OTUs were obtained in the C, LD, MD, and HD groups, respectively. With an increase in the fried oil dose, the OTU values of the gut microbiota in mice gradually decreased. These results indicate that a certain dose of fried oil can reduce the species diversity of the gut microbiota in mice in the short term. The total OUT counts in the LD, MD, HD, and C groups were 2845, 2771, and 2689, respectively. As the dosage of fried oil increased, the population of OTUs shared in the gut microbiota of mice treated with fried oil and non-fried oil gradually declined, indicating an increasing divergence in the gut microbiota between the two groups. A principal coordinate analysis using UniFrac indicated differences in the gut microbiota between samples and revealed different clusters of microbiota composition in each group. As illustrated in Figure 3B, the differences in the gut microbiota between samples were insignificant, indicating that the structural characteristics of the gut microbiota were analogous in all groups. In Figure 3C, the Shannon index of the MD and HD groups was significantly lower than that of the C group (*p* < 0.05).

In conclusion, this indicates that in terms of alpha diversity, intake of medium and high doses of fried oil can reduce the richness and diversity of the gut microbiota.

### 3.4. Effects of Fried Oil on the Abundance of Gut Microbiota at the Phylum Level

**Figure 4:**
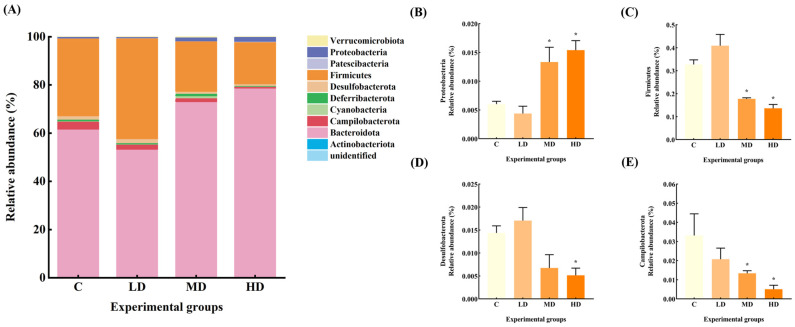
(**A**) Abundance plots of species composition at the phylum level for each group of mice gut microbiota. (**B**) Proteobacteria relative abundance. (**C**) Firmicutes relative abundance. (**D**) Desulfobacterota relative abundance. (**E**) Campilobacterota relative abundance. Data are expressed as mean ± SD. * *p* < 0.05 vs. C.

The gut microbiota constitutive abundance of the mice in each group at the phylum level is shown in Figure 4. The relative abundances of gut microbiota Firmicutes and Desulfobacterota in the LD group increased by 25% and 19%, respectively, compared to the C group. The relative abundance of Deferribacterota and Campilobacterota decreased by 31% and 37%, respectively.

The relative abundance of Proteobacteria in the gut microbiota of mice in the MD group increased by 122% (*p* < 0.05), and that of Deferribacterota increased by 27%. The relative abundance of Firmicutes and Campilobacterota decreased by 46% and 60%, respectively (*p* < 0.05), and Desulfobacterota decreased by 53%.

The relative abundance of Proteobacteria in the HD group was significantly increased by 156% (*p* < 0.05), whereas the relative abundance of Desulfobacterota, Firmicutes, and Campilobacterota was significantly decreased by 64%, 58%, and 85%, respectively (*p* < 0.05).

### 3.5. Effects of Fried Oil on Gut Microbiota Abundance at Genus Level

**Figure 5:**
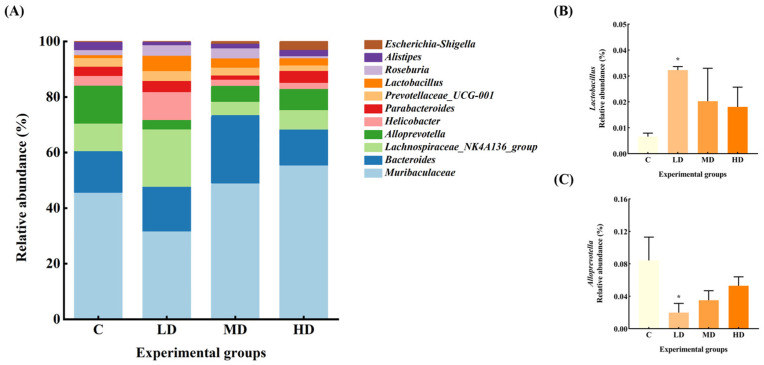
(**A**) Abundance plots of species composition at the genus level for each group of mouse gut microbiota. (**B**) *Lactobacillus* relative abundance. (**C**) *Alloprevotella* relative abundance. Data are expressed as mean ± SD. * *p* < 0.05 vs. C.

The gut microbiota of the mice in each group at the genus level are shown in Figure 5. The relative abundance of *Muribaculaceae* and *Alistipes* in the gut microbiota of mice in the LD group was reduced by 37% and 60%, respectively, compared with the C group. *Alloprevotella* significantly decreased by 76% (*p* < 0.05). The relative abundance of *Lachnospiraceae_NK4A136_group*, *Helicobacter*, and *Roseburia* increased by 96%, 170%, and 123%, respectively. Notably, the abundance of Lactobacillus significantly increased by 385% (*p* < 0.05).

The relative abundance of *Escherichia–Shigella* in the MD group increased 8-fold, and *Bacteroides*, *Roseburia*, *Lactobacillus*, and *Muribaculaceae* by 62%, 95%, 207%, and 6%, respectively, whereas the relative abundance of *Lachnospiraceae_NK4A136_group*, *Alloprevotella*, *Parabacteroides*, and *Alistipes* decreased by 52%, 58%, 56%, and 42%, respectively.

The relative abundance of *Escherichia–Shigella* increased by 39-fold in the HD group. *Parabacteroides*, *Muribaculaceae*, and *Lactobacillus* increased by 45%, 37%, and 174%, respectively, while the abundance of *Bacteroides*, *Helicobacter*, *Alloprevotella*, and *Alistipes* decreased by 3%, 29%, 37%, and 13%, respectively.

### 3.6. Effects of Fried Oil on Fecal SCFAs

**Figure 6:**
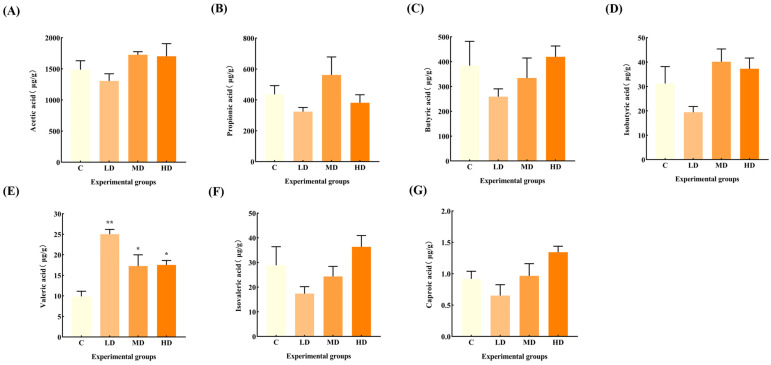
Effects of fried oil on the SCFA concentration in mice feces. (**A**) Acetic acid. (**B**) Propionic acid. (**C**) Butyric acid. (**D**) Isobutyric acid. (**E**) Valeric acid. (**F**) Isovaleric acid. (**G**) Caproic acid. Data are expressed as mean ± SD. * *p* < 0.05, ** *p* < 0.01 vs. C.

Figure 6 reveals that there was no significant change in the levels of acetic acid, propionic acid, butyric acid, isobutyric acid, isovaleric acid, and caproic acid in the feces of mice in the fried oil group compared with the C group, but the levels of valeric acid were significantly increased by 153% (*p* < 0.01), 89%, and 78%, respectively (*p* < 0.05).

### 3.7. Correlation Analysis of Gut Microbiota, SCFA Concentration, and TNF-α Levels

**Figure 7:**
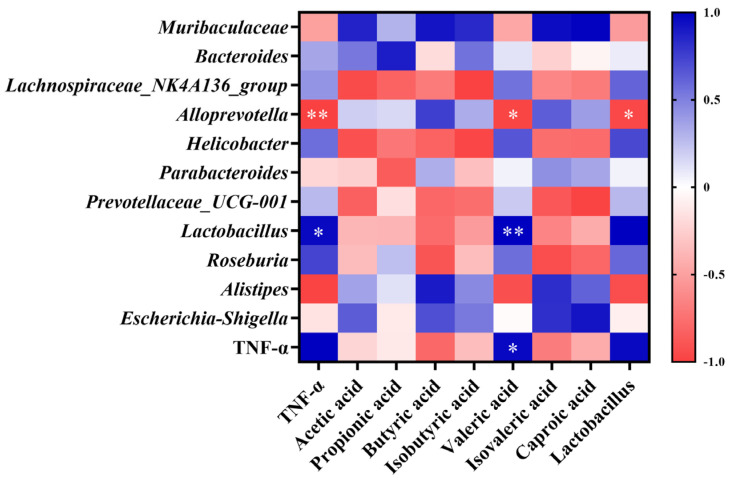
Pearson correlation analysis of gut microbiota structure, SCFA concentration, and TNF-α levels. Data are expressed as mean ± SD. * *p* < 0.05, ** *p* < 0.01.

As shown in Figure 7, *Alloprevotella* abundance was significantly negatively correlated with TNF-α levels (*p* < 0.01). *Lactobacillus* abundance was significantly positively correlated with TNF-α levels (*p* < 0.05). Valeric acid concentration was significantly positively correlated with TNF-α levels (*p* < 0.05) and *Lactobacillus* abundance (*p* < 0.01), and significantly negatively correlated with *Alloprevotella* abundance (*p* < 0.05). *Lactobacillus* abundance was significantly negatively correlated with the abundance of *Alloprevotella* (*p* < 0.05).

## 4. Discussion

Previous research indicates that obese individuals with lower bacterial richness gain more weight over time [10]. Additionally, a prolonged consumption of high-fat diets can also alter the gut microbiota composition, leading to an increased inflammatory state and obesity [11]. In the current study, a significant increase in body weight was observed in mice in the LD, MD, and HD groups compared to the C group, suggesting that even a short-term intake of a certain dose of fried oil can significantly increase body weight, while systemic low-grade inflammation can contribute to obesity.

Among the pro-inflammatory cytokines, TNF-α can mediate macrophages to release cytokines, causing low-grade inflammation and apoptosis [12]. Long-term intake of fried oil can increase plasma pro-inflammatory cytokines, but short-term intake of fried oil significantly increased by two to four times in body systems is rarely reported [13]. In this study, serum TNF-α was significantly increased by two to four times in mice administered fried oil within a week compared to that in the C group.

The Shannon index analysis showed that the richness and diversity of gut microbiota in the MD and HD groups were significantly reduced at 6d, which is consistent with the results of a previous study in mice fed fried corn oil and lard for six weeks [14].

The relative abundance of Proteobacteria is associated with toxins, which contain a variety of pathogenic bacteria (such as *Escherichia*, *Shigella*, etc.) that mediate cytokine aggregation and the formation and exacerbation of inflammation, which is a potential diagnostic indicator of ecological disorders and disease risk, and an increase in the abundance of Proteobacteria may further facilitate invasion by exogenous pathogens [15]. In intestinal inflammatory syndromes, such as irritable bowel syndrome (IBS) and metabolic syndrome, an increase in the abundance of Proteobacteria has been evident [16]. In our study, the relative abundance of Proteobacteria in gut microbiota of mice in the MD and HD groups was significantly increased by 122% and 156%, respectively, with the relative abundance of *Escherichia–Shigella* increased by 8 and 39 times, respectively. Furthermore, the abundance of *Lactobacillus* in the LD group was significantly increased, whereas that of *Alloprevotella* significantly decreased. All these factors point to a disturbance in the gut microbiota upon ingesting fried oil, even in the short term.

Lactic acid bacteria can aid in balancing the gut microbiota and boosting immunity. *Lactobacillus* converts lactose into lactic acid, which is an important metabolic intermediate in SCFA metabolism but can also serve as a fuel source for cancer cells, promoting inflammation, angiogenesis, and metastasis [17]. The immunomodulatory effect of *Lactobacillus* can also be achieved through the release of cytokines induced by its metabolites, including SCFAs, tumor necrosis factor, interferon, and transforming growth factor [18]. A long-term high-fat diet can lead to a reduced number of *Lactobacilli* in the gut; conversely, *Lactobacillus* can alleviate high-fat diet-induced obesity in mice [19,20]. However, in our study, the relative abundance of *Lactobacillus* in the LD group significantly increased by up to 385% in the short term. According to the Pearson correlation analysis, the relative abundance of *Lactobacillus* was significantly positively correlated with TNF-α levels. This result contradicts the common belief that an increase in *Lactobacillus* abundance can reduce the levels of inflammatory factors. It seems that an increase in *Lactobacilli* does not necessarily mean better health, and in fact a large increase in *Lactobacillus* abundance may promote an increase in inflammatory factor levels [21,22].

*Alloprevotella* can decompose protein- and carbohydrate-containing foods. It can also function as a conditional pathogen that induces intestinal inflammation. *Alloprevotella* is more abundant in vegetarians compared with non-vegetarians [23], and is generally considered to be a bacterium associated with a healthy plant-based diet, acting as a “probiotic” in the human body and therefore any reduction can lead to disease [24]. Recent human studies have linked increased *Alloprevotella* abundance to local and systemic low-grade inflammation [25]. In our study, *Alloprevotella* abundance decreased significantly in the LD group and was significantly negatively correlated with *Lactobacillus* abundance and TNF-α levels, suggesting that frying increased soybean oil acidity, making the intestinal environment more favorable for *Lactobacillus* growth. The proliferation of *Lactobacillus* produces metabolites such as lactic acid and valeric acid, which further increase gut acidity and inhibit the growth, reproduction, and activity of *Alloprevotella*, culminating in a reduced abundance [26]. It has been suggested that the decrease in *Alloprevotella* abundance may indirectly contribute to reducing the inflammatory response and promoting gut ecological balance in the context of fried oil intake. In some studies, in rats fed fried soybean oil for six weeks, it was found that gut *Lactobacillus* abundance was significantly increased and *Alloprevotella* abundance was significantly decreased, which is consistent with our study. It is speculated that the immune system has the capacity to suppress multiple oxidative stress products formed during feeding of fried oil by promoting the proliferation of *Lactobacillus*, whereas the decrease in *Alloprevotella* abundance is related to the fermentation and utilization of carbohydrates and monosaccharides [27].

Most previous studies on the induction mechanism of systemic low-grade inflammation have primarily focused on other signaling pathways such as nuclear factor kappa-B (NF-κB) and mitogen-activated protein kinase (MAPK). However, research has linked gut microbiota metabolites and SCFAs to act as anti-inflammatory agents via G protein-coupled receptors and histone deacetylase [28,29]. Recent research has indicated that valeric acid, a branched short-chain fatty acid, is negatively correlated with good health. Excessive blood valerate concentration exacerbates inflammation and damages brain cells and dendritic branches; however, gut microbiota changes and elevated blood valerate levels can be alleviated by appropriate exercise [30]. The fecal valeric acid concentration in children with autism is higher than that of acetic acid and butyric acids [31]. A significant increase in fecal valeric acid levels occurs in patients with IBS, and fecal valeric acid levels are significantly reduced following treatment [32], suggesting that an excessive accumulation of valeric acid in the gut is detrimental to health. The Pearson correlation analysis showed a significant positive correlation between fecal valeric acid and TNF-α levels, which suggests that fried oil consumption affects health, even in the short term. Thus, an increase in fecal valeric acid levels, a metabolic product of the gut microbiota, increases TNF-α levels, and induces low-grade inflammation in the body. This result also provides an important theoretical basis for the search for control targets for systemic low-grade inflammation induced by fried oil.

This study indicated that fried oil, even at a low concentration and for a short duration, can cause gut microbiota changes. Such disturbances can result in the overgrowth of *Lactobacillus* and a reduction in the proliferation of *Alloprevotella*. Subsequently, there was an increase in the bacterial metabolite valeric acid, contributing to a significant increase in serum TNF-α levels. However, upon exposure to a high concentration of fried oil, TNF-α declined, but the gut microbiota metabolites isovaleric acid, butyric acid, and caproic acid showed an increasing trend with minimal changes in SCFAs. Notably, this phenomenon may stem from the non-linear dose–response relationships that are common in biological systems. Within a certain range, increasing exposure levels may attenuate, rather than enhance, the strength of an organism’s response, reflecting the complex regulatory mechanisms in the body, including adaptive responses and negative feedback inhibitory pathways [33]. For example, initial exposure to low doses of fried oil may stimulate a rapid TNF-α response; however, the subsequent activation of negative feedback mechanisms may have led to a decrease in its levels at the time point of our observations [34]. In addition, our study focuses on short-term exposure effects, where organisms may utilize adaptive mechanisms to respond effectively to such inflammatory stimuli, explaining the observation of a short-term decline in TNF-α. However, long-term exposure scenarios, where these adaptive mechanisms may gradually fail and cumulative damage exceeds repair capacity, may eventually manifest as a persistent or exacerbated inflammatory response, leading to increased TNF-α levels, a direction worthy of further exploration in future studies [35]. Previous reports have shown that an increase in gut butyric acid concentration induces an inhibitory effect on TNF-α levels [36]. Whether butyric acid influences TNF-α levels can be tested by extending the test period in future studies. A future goal would be to regulate gut microbiota to stimulate the growth of specific microorganisms, thereby increasing the proportion of target gut microbiota or finding a substance that can inhibit the increase in the gut metabolite valeric acid, thereby minimizing inflammation in the body. According to our study, consuming a certain dose of fried oil even for a short period can lead to an increase or decrease in selective gut microbiota, resulting in an imbalance in microbiota homeostasis and an increase in fecal valeric acid and inflammatory factor TNF-α in serum, inducing a low-degree inflammation in the body.

## 5. Conclusions

The consumption of fried oil, even for a short period, promoted the growth of *Lactobacillus* and inhibited *Alloprevotella*, leading to an imbalance in the gut microbiota. Gut dysbiosis was associated with a significant increase in fecal valeric acid and a significant increase in serum TNF-α levels by up to 4-fold, both of which induced systemic low-grade inflammation in mice. Future studies should consider higher levels of evidence, such as clinical observations or human interventional studies, to validate and extend the current results observed in mouse models. In summary, this study provides a theoretical basis for evaluating the effects of short-term intake of fried oil on human health and to identify a compound that can mitigate the upsurge in inflammation in the body caused by the consumption of fried oil in the diet.

## Data Availability

The raw data supporting the conclusions of this article will be made available by the authors upon request.
